# The TIF-1γ autoantigen in patients with dermatomyositis and thyroid cancer: from case series to preliminary mechanistic exploration

**DOI:** 10.3389/fimmu.2026.1817558

**Published:** 2026-09-08

**Authors:** Xiayan Xu, Yichi Zhang, Zixuan Huang, Ning Shen, Ruixia Tang, Mingli Ouyang, Linrong Lu, Yongmei Han

**Affiliations:** 1 Department of Rheumatology, Sir Run Run Shaw Hospital, Zhejiang University School of Medicine, Hangzhou, Zhejiang, China; 2 Zhejiang University-University of Edinburgh Institute (ZJU-UoE Institute), Zhejiang University School of Medicine, Zhejiang University, Haining, China; 3 School of Physics, Peking University, Beijing, China; 4 Dr. Li Dak Sum & Yip Yio Chin Center for Stem Cell and Regenerative Medicine, Zhejiang University School of Medicine, Hangzhou, China; 5 Shanghai Immune Therapy Institute, Renji Hospital, Shanghai Jiao Tong University School of Medicine, Shanghai, China; 6 Liangzhu Laboratory, Zhejiang University Medical Center, Hangzhou, China

**Keywords:** cancer associated dermatomyositis, dermatomyositis, thyroid cancer, TIF1γ, TIF1γ-positive dermatomyositis

## Abstract

**Objectives:**

This study aimed to explore the relationship between dermatomyositis (DM) and thyroid cancer and the role of transcriptional intermediary factor 1γ (TIF-1γ) in the pathogenesis of cancer-associated DM.

**Methods:**

Five cases of DM with thyroid cancer, including one with metastasis, were summarized and analyzed. Published cases of DM with thyroid cancer were reviewed. Subsequently, a bidirectional two-sample Mendelian randomization (MR) analysis was performed to investigate the relationship between thyroid cancer and myositis using inverse-variance weighting (IVW), MR-Egger, and weighted-median (WM) analyses. Finally, immunohistochemistry (IHC) and immunofluorescence (IF) analysis were performed to detect TIF-1γ in tumor tissues of a DM patient and a thyroid cancer patient without DM.

**Results:**

This article reports the first case of a patient with DM and metastatic papillary thyroid carcinoma (PTC), in whom recurrence of the rash prompted the detection of metastasis, with the rash resolving after surgical resection of the metastatic foci. MR analysis showed a potential causal relationship between thyroid cancer and myositis (OR = 1.398; 95% CI = 1.045–1.869; *p* = 0.029). A higher expression level of TIF-1γ was observed in tumor tissues of DM patients compared with those of thyroid cancer patients without DM by immunofluorescence (IF) analysis (*p* < 0.0001).

**Conclusions:**

DM symptoms might represent a paraneoplastic phenomenon in patients with thyroid cancer, with the underlying pathological mechanisms requiring further exploration and TIF-1γ overexpression potentially serving as a trigger.

## Introduction

Dermatomyositis (DM) is an idiopathic inflammatory myositis (IIM) with high heterogeneity, characterized mainly by chronic inflammation of the muscle and skin. Other systemic involvements are also common in patients with DM, including interstitial lung disease, arthritis, and gastrointestinal and cardiac diseases (1). Adult-onset DM is associated with a high risk of malignancy, with standardized incidence ratios (SIRs) ranging from 2.17 to 6.5 (2), suggesting a potential correlation between DM and cancer. DM patients with cancer have a poor prognosis, with cancer being one of the leading causes of death. Antitranscriptional-intermediary-factor-1γ (anti-TIF-1γ) antibody is a classic marker for DM and is reported to be associated with an increased risk of malignancy in DM patients (3). However, the underlying mechanisms remain unclear, among which the paraneoplastic hypothesis is the most frequently cited (4). This article reports the first case of a patient with DM and metastatic papillary thyroid carcinoma (PTC), in whom recurrence of the rash prompted the detection of metastasis, with the rash resolving after surgical resection of the metastatic foci. Using Mendelian randomization (MR), immunohistochemistry (IHC), and immunofluorescence (IF) analyses, we further confirmed the relationship between myositis and thyroid cancer, as well as the role of transcriptional intermediary factor 1γ (TIF-1γ).

## Materials and methods

### Patients

The study included all adult DM patients with thyroid cancer in Sir Run Run Shaw Hospital, School of Medicine, Zhejiang University (Zhejiang, China), between February 2017 and December 2025 who fulfilled the 2017 EULAR/ACR IIM classification criteria for DM (5). The study was approved by the ethics committee of Sir Run Run Shaw Hospital, School of Medicine, Zhejiang University, and all participants gave written informed consent.

### MR analysis

We performed a bidirectional two-sample MR analysis to explore the causal associations between thyroid cancer and myositis based on the largest genome-wide association study (GWAS) database. This MR study was conducted according to the guidelines of the STROBE-MR Statement (6). This MR analysis relied on three core assumptions: (1) the genetic variants were strongly associated with the exposure (thyroid cancer or myositis); (2) the genetic variants were independent of any confounders that might affect the relationships between thyroid cancer and myositis; and (3) the genetic variants influenced the outcome only through their effect on the exposure and not through alternative pathways.

To ensure the robustness of these genetic instrumental variables (IVs), stringent selection criteria were used: (1) only single nucleotide polymorphisms (SNPs) that reached genome-wide significance (*p* < 5 × 10^−8^) and displayed independence as determined through linkage disequilibrium (LD, *r^2^* < 0.1) were considered; and (2) SNPs with an *F*-statistic > 10 were considered to reduce bias resulting from weak IVs. The selected SNPs were subsequently employed as IVs for the two-sample MR analysis. The details of the GWASs are shown in **Table 1**.

**Table 1:** Details of the GWAS used in the Mendelian randomization.

Trait	Sample size	Source
Thyroid cancer	1,620,354	https://ftp.ebi.ac.uk/pub/databases/gwas/summary_statistics/GCST90399001-GCST90400000/GCST90399737/
Myositis	456,348	https://ftp.ebi.ac.uk/pub/databases/gwas/summary_statistics/GCST90044001-GCST90045000/GCST90044617/

### IHC and IF analysis

Tumor samples were obtained from Sir Run Run Shaw Hospital. One sample was metastatic lymph node tissue from a DM patient with PTC described in the case report, and the other was also metastatic lymph node tissue from a gender- and age-matched PTC patient without DM. Tissue sections were prepared by the Department of Pathology at Sir Run Run Shaw Hospital. Hematoxylin and eosin (H&E) staining was performed by the Core Facilities of Zhejiang University School of Medicine.

Immunofluorescence for TIF-1γ protein was performed using the tyramide signal amplification (TSA) method. First, sections were deparaffinized in xylene and rehydrated through a graded ethanol series. Antigen retrieval was performed in EDTA buffer (pH 9.0) using microwave heating. Endogenous peroxidase activity was quenched with 3% H_2_O_2_, followed by blocking with 3% bovine serum albumin. Sections were incubated overnight at 4 °C with a rabbit monoclonal anti-TIF-1γ primary antibody (CST No. 90051) diluted to 1:1,000. After washing, a horseradish peroxidase (HRP)-conjugated goat antirabbit/mouse universal secondary antibody was applied. Signal amplification was achieved using a TSA fluorophore working solution (TYR-570 Plus, diluted 1:200 in TSA buffer), which was incubated for 10 min in the dark. Nuclei were counterstained with DAPI, and tissue autofluorescence was reduced using an autofluorescence quenching reagent. Slides were mounted with antifade medium.

### Statistical analyses

The three principal analysis methods used in MR studies were MR-Egger, inverse-variance weighting (IVW), and weighted-median (WM) methods. A sensitivity analysis was performed in our study to ensure the robustness of the results. The heterogeneity of each SNP was evaluated using Cochran’s *Q* statistic, with a *p*-value < 0.05 indicating significant heterogeneity among the estimates of the included SNPs.

Continuous variables were compared using Student’s *t*-test. Statistically significant differences between patients were assessed using a two-tailed Student’s *t*-test. *p* < 0.05 was considered statistically significant. All statistical analyses were performed using SPSS software (version 26.0; SPSS Inc., Chicago, IL, USA).

## Results

### Case review

**Figure 1:**
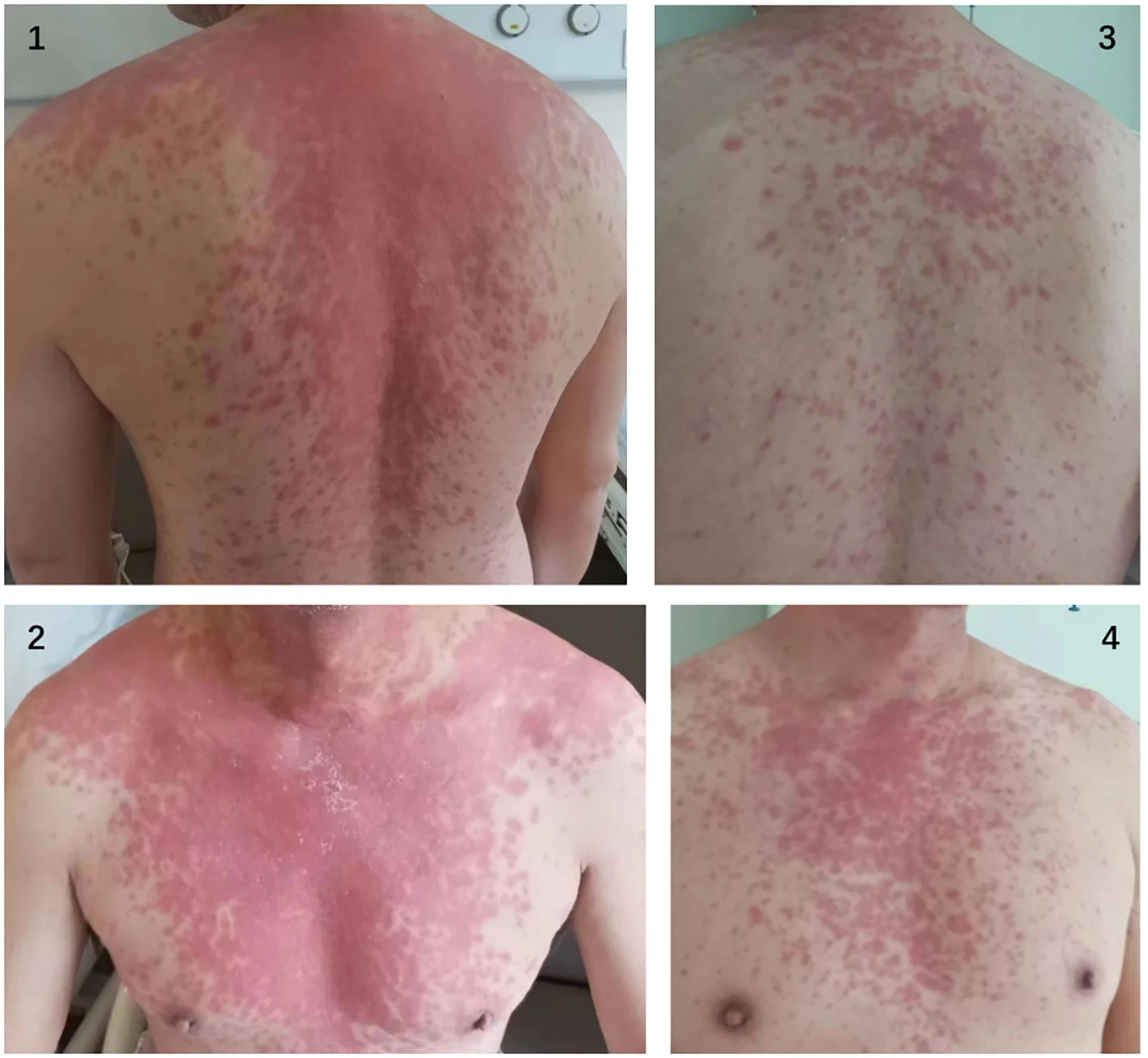
Rash over different periods. (1, 2) One week before functional cervical lymph node dissection (V-sign and shawl sign, respectively). (3, 4) One week after functional cervical lymph node dissection (V-sign and shawl sign, respectively).

In August 2022, a 47-year-old man with a 4-month history of recurrent rash was admitted to our hospital. Four months earlier, the patient developed a red rash around the eyes without any obvious triggers, with occasional itching. He was first diagnosed with eczema at a local hospital, with no improvement in symptoms after antiallergic treatment. Subsequently, he visited another hospital, where the anti-TIF-1γ antibody was detected as positive. Further physical examination showed bilateral thigh weakness, with MRI showing abnormal signals in the superficial muscle groups and myofascial spaces of both thighs, as well as in the skin and subcutaneous connective tissue of the buttocks and thighs on both sides. He was then diagnosed with DM, with his condition improving after treatment with methylprednisolone at an initial dose of 40 mg/day, followed by gradual tapering. One month earlier, the patient again developed a symmetrical rash on the face, anterior chest, posterior back, and both hands and feet, with obvious itching. Subsequently, he was successively given dexamethasone 10 mg twice a day, dexamethasone 20 mg twice a day, methylprednisolone 120 mg twice a day, and was gradually transitioned to methylprednisolone 80 mg once a day, combined with hydroxychloroquine 200 mg twice daily. During this period of glucocorticoid tapering, the rash persisted with recurrent episodes and minimal regression. Therefore, he came to our hospital for further treatment. Notably, history taking revealed that the patient underwent bilateral thyroidectomy due to thyroid cancer on 25 October 2021. Considering the close relationship between DM and cancer, this patient received a PET-CT examination while receiving glucocorticoid and hydroxychloroquine therapy combined with human immunoglobulin therapy. PET-CT showed that the bilateral thyroid glands were absent, with a nodular shadow anterior to the trachea (above the level of the sternal manubrium) showing increased FDG metabolism, and metastasis could not be excluded. Further thyroid ultrasound showed postoperative changes in the thyroid bed, with an enlarged lymph node in the suprasternal fossa. Considering the poor response to glucocorticoids and the detection of anti-TIF-1γ antibody, cancer recurrence or metastasis could not be excluded. Thus, endoscopy-assisted cervical lymph node dissection was performed on 3 September, which revealed metastatic papillary thyroid cancer in a lymph node of the suprasternal fossa. Following surgery, the patient showed marked regression of the rash with regular methylprednisolone tapering (**Figure 1**). Thereafter, the patient underwent regular follow-up in our hospital. At the latest visit in January 2025, the patient came to our department with no obvious rash and was maintained on methylprednisolone 4 mg once daily and hydroxychloroquine 100 mg twice daily. No evidence of lymph node enlargement was observed on cervical ultrasonography.

### Characteristics of DM with thyroid cancer

Subsequently, four cases of DM with thyroid cancer at Sir Run Run Shaw Hospital from February 2017 to December 2025 were included in this study (**Table 2**). Two of the four patients were women (50%), and the mean age was 46 years ± 1.8 years. Myositis-specific autoantibodies (MSAs) were diverse, and only one patient was anti-TIF-1γ positive (the one reported in this article). Most patients (three of four, 75%) were diagnosed with PTC. All patients were found to have cancer within 3 years of a definite diagnosis of DM, indicating the importance of close screening and monitoring for thyroid cancer in DM populations. Interstitial lung disease (ILD) was present in 50% of patients (two of four). Half of the patients were diagnosed with amyopathic DM (ADM) with normal CK levels. Only one case (the one reported in this article) was found to have metastasis during follow-up, partly due to the fact that the longest follow-up period was only 3 years and some patients were lost to follow-up. All patients underwent surgical resection of thyroid cancer; two showed dramatic improvement afterward and were maintained with low-dose steroids (one case) or without any medication (one case). The other two cases were diagnosed with cancer before DM, with no obvious correlation between cancer therapy and DM.

**Table 2:** The demographic characteristics of the four cases.

Sex	Age at diagnosis	MSAs	CK level	ILD	Type of cancer	Temporal relationship between the diagnoses	Follow-up
M	44	MDA5	High	Yes	PTC	At the same time	3 years without relapse
M	47	TIF-1γ	Normal	No	PTC	PTC diagnosis 8 months before DM	Metastasis 10 months later
F	48	MDA5	Normal	Yes	Not clear	Cancer diagnosis 2 years before DM	Nearly 2 years without relapse
F	45	Mi-2α, Mi-2β	High	No	PTC	PTC diagnosis 7 months before DM	1 year without relapse

MSAs, myositis-specific autoantibodies; CK, creatine kinase; ILD, interstitial lung disease; M, male; F, female; PTC, papillary thyroid carcinoma.

### Mendelian randomization analysis showed a potential causal relationship between thyroid cancer and myositis

**Figure 2:**
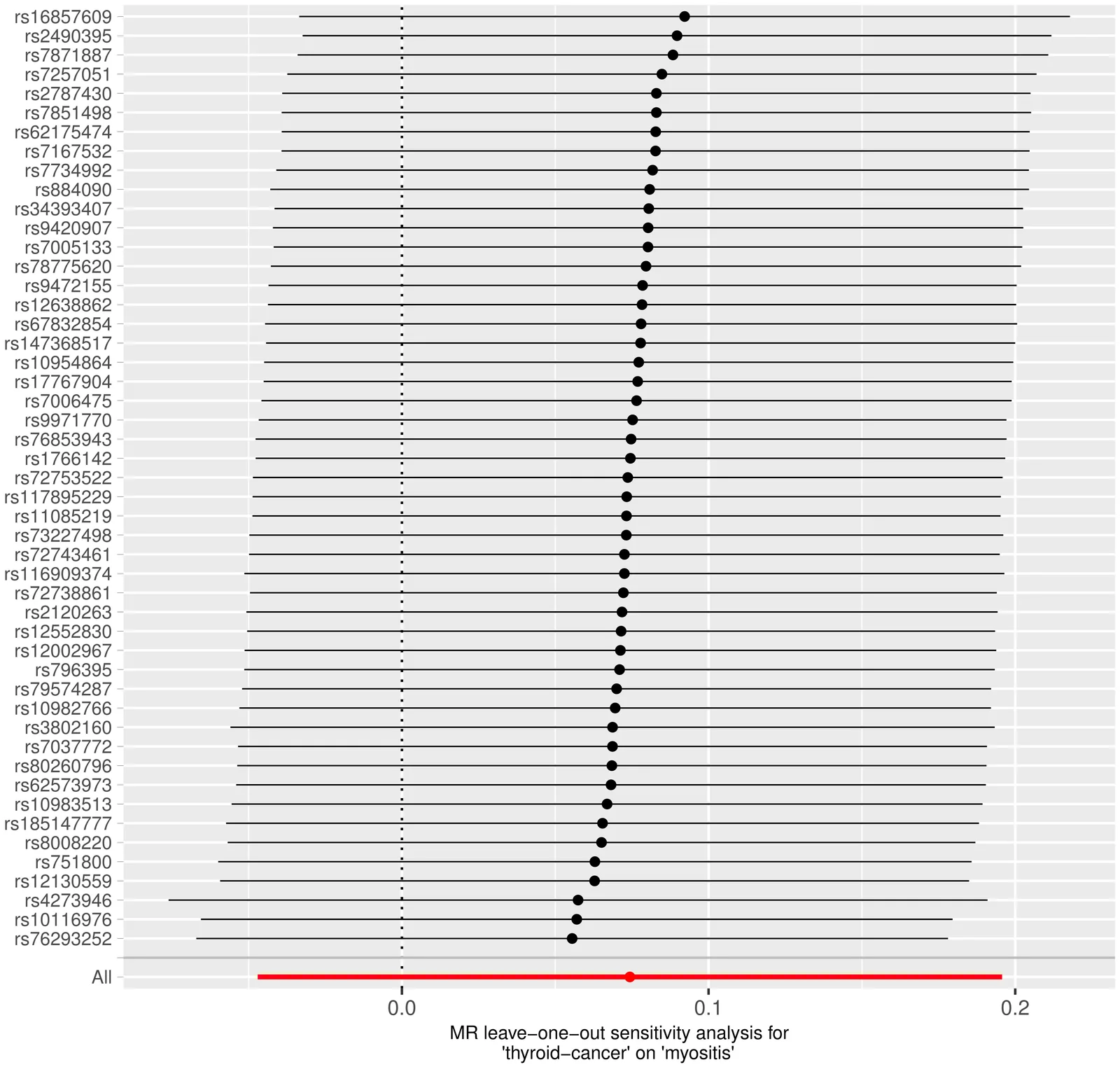
MR leave-one-out sensitivity analysis for thyroid cancer and myositis.

The MR-Egger estimate showed a potential causal association between thyroid cancer and myositis risk (odds ratio [OR] = 1.398; 95% confidence interval [CI] = 1.045–1.869; *p* = 0.029) (**Table 3**), while no such association was found with the WM (OR = 1.077; 95% CI = 0.954–1.261; *p* = 0.230) or the IVW method (OR = 1.142; 95% CI = 0.935–1.394; *p* = 0.193) (**Table 3**). The leave-one-out test suggested that the MR analysis results were reliable (**Figure 2**). On the other hand, no causal effects of myositis on thyroid cancer were detected by MR-Egger (OR = 1.0005; 95% CI = 0.984–1.027; *p* = 0.628), the WM (OR = 1.002; 95% CI = 0.985–1.020; *p* = 0.810), or the IVW method (OR = 1.000; 95% CI = 0.987–1.013; *p* = 0.984) (**Supplementary Table 2**). However, whole-exome sequencing (WES) of this patient showed no clinically relevant mutation.

**Table 3:** MR estimates and sensitivity analyses for associations of thyroid cancer and risk of myositis.

nSNPs	Method	OR	95% CI	Forest plot	*p*-value	*Q*_*p*-value
49	MR-Egger	1.398	1.045–1.869	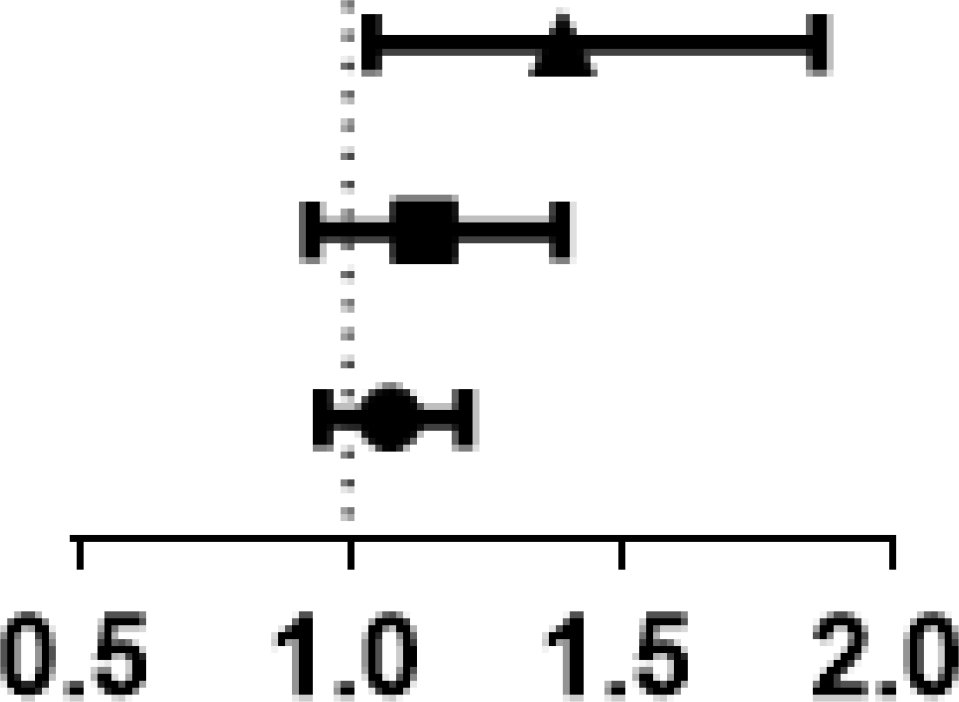	0.029	0.73
49	Weighted-median	1.142	0.935–1.394		0.193	
49	Inverse-variance-weighted	1.077	0.954–1.261		0.230	0.62

SNP, single nucleotide polymorphism.

### Higher deposition of TIF-1γ in the tumor tissue of the DM patient

**Figure 3:**
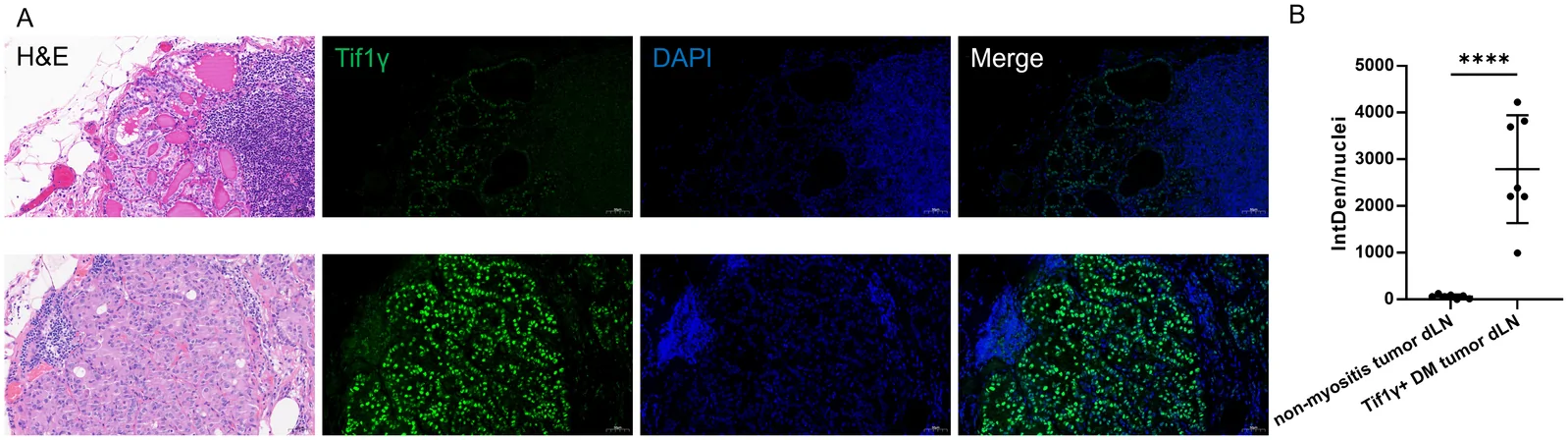
Higher deposition of TIF1-γ protein was detected in the metastatic lymph node tissue of a DM patient. **(A)** Representative immunohistochemical staining with H&E and immunofluorescence costaining of TIF-1γ with DAPI. **(B)** Each dot represents one field of view. Horizontal lines indicate the patient mean ± SD (^****^p < 0.0001, by unpaired *t*-test).

Next, we investigated the expression level of TIF-1γ protein in the metastatic lymph node tissue from the patient in this case, compared with that in a gender- and age-matched PTC patient without DM. Higher deposition of TIF-1γ protein was detected in the DM patient (**Figure 3**; *p* < 0.0001), which suggested that TIF-1γ might serve as an initiating factor for the development of DM in cancer patients.

## Discussion

DM is an idiopathic inflammatory disease with characteristic skin and muscle manifestations that is highly associated with neoplasms. A prevalence of 14.8% was recently reported for tumors in DM patients (7). Interestingly, some patients may recover from DM after appropriate treatment of the tumor, implying a paraneoplastic phenomenon. Yeh et al. (8) summarized eight cases of DM with breast cancer, which is one of the most common types of cancer in DM patients (9), and found that four of five patients had parallel improvement after surgical resection, with three ultimately achieving disease-free survival. Ovarian cancer is also common in DM patients, particularly in women, with a prevalence of 13.3%–26% (10). Consistently, DM manifestations resolved after treatment for ovarian cancer, in parallel with the decrease in CA125 levels (11–13). However, some articles have reported different opinions. In fact, there have been cases in which DM remained refractory even after successful cancer treatment (14). On the other hand, the high risk of malignancy even 5 years after the first diagnosis of myositis also challenged the concept of the paraneoplastic phenomenon (15). In this case, our patient was diagnosed with DM with a 10-month history of thyroid cancer. Meanwhile, he was found to have metastatic disease on PET-CT and underwent surgery, after which the rash and other DM symptoms resolved, supporting the paraneoplastic hypothesis.

Correspondingly, exacerbation of DM manifestations sometimes indicates recurrence or metastasis of cancer (16). Takahashi et al. (16) found that cancer relapses could be detected by aggravation of DM symptoms, especially when a severe rash suddenly spread all over the body. They also found that such patients might have a poor response to surgery, as three of five cases died of this disease after surgery. This conclusion requires more data to support it and may facilitate a better understanding of the need to combine surgery with other treatments, including chemoradiotherapy and DM therapy, as early as possible (16). Also, recurrence of DM symptoms could be the first sign indicating relapse of ovarian cancer (11). In our case, the patient first presented with an aggravated rash after regular treatment for DM, which was finally confirmed as metastatic thyroid cancer. Thus, it is important to remain alert to DM symptoms in patients with a history of cancer to exclude cancer recurrence or metastasis.

TIF-1γ protein is known to be involved in various biological processes, including cell proliferation and differentiation, immune response, and tumorigenesis (17). Antibodies against this protein (anti-TIF-1γ) were first identified in 2006 (18) and gradually recognized as a kind of MSA. Importantly, anti-TIF-1γ was reported to be closely related to an increased risk of cancer in DM patients, with a prevalence of up to 23% (19). Up to now, the underlying pathogenetic mechanisms of the high cancer risk in anti-TIF-1γ-positive DM remain unclear. TIF-1γ is now considered possibly a tumor autoantigen in the pathogenesis of DM (17). The higher expression level of TIF-1γ protein in the tumor tissue of DM patients compared with that in the tumor tissue of patients without DM also supported this hypothesis. On this basis, we further speculated that, in DM patients accompanied by cancer, TIF-1γ protein is highly expressed in tumor tissues and is massively released after antitumor responses, thereby inducing the production of anti-TIF-1γ antibodies that target normal tissues, including skin and muscle, and finally leading to the development of DM. This hypothesis needs to be confirmed in broader populations as well as in animal models.

Thyroid cancer is a special kind of tumor with low incidence (20). PTC accounts for about 80% of cases and is characterized by low rates of metastasis and a good prognosis. Mortality from PTC is also reported to be less than one per 100,000 (21). Similarly, reported cases of DM with thyroid cancer are very rare (22). However, different studies have reported differing opinions about the relationship between DM and thyroid cancer (23, 24). Meanwhile, the incidence of thyroid cancer, especially PTC, has increased rapidly in the past few years (25). Thyroid cancer was the ninth most common cancer worldwide in 2020 (26). In our DM cohort of 154 patients from February 2017 to December 2025, four cases (2.60%) were found to have concurrent thyroid cancer. This observed prevalence is substantially higher than the estimated lifetime cumulative risk of thyroid cancer in the general Chinese population, which is approximately 0.5%–0.6% based on GLOBOCAN 2022 data (27). Therefore, it is meaningful to further explore DM with thyroid cancer. However, given the limited number of cases, this result should be interpreted with caution. Furthermore, the potential for surveillance bias, whereby DM patients may undergo more frequent imaging examinations, cannot be excluded as a contributing factor to the higher observed detection rate.

Subsequently, we searched PubMed using keywords “thyroid cancer” and “dermatomyositis” in December 2025. Thirteen cases were included (**Supplementary Table 1**), with women accounting for 76.9% (10/13), consistent with the gender distribution of thyroid cancer. The mean age was 53.7 years ± 10.4 years, with PTC being the most common type (12/13). MSA data were missing in most cases due to technical constraints, with anti-TIF-1γ and anti-MDA5 each being positive in one case. More than half of the cases (seven of 13, 53.8%) had muscle involvement, and 30.8% (four of 13) were accompanied by ILD. Most cases (12/13, 92.3%) were found to have cancer within 3 years of a definite diagnosis of DM, especially during the first year (11/13, 84.6%), with no case showing recurrence or metastasis, which was consistent with the general condition in thyroid cancer patients but contrary to the findings of this article. Only three cases reported a definite good response to antitumor therapy, while one case even showed exacerbation of DM symptoms after cancer surgery (28). Comparing the features of patients with DM and thyroid cancer in our center with those reported in the published articles, it was obvious that the first year after DM diagnosis represented a period of high risk for thyroid cancer. Thus, thorough examinations and regular monitoring during follow-up are of high importance. Meanwhile, even though thyroid cancer has a low frequency of metastasis, a detailed assessment of DM and cancer, including PET-CT when appropriate, should be performed promptly if DM symptoms relapse. However, the relationship between DM and thyroid cancer remains unclear, as not all cases showed improvement in DM symptoms after antitumor therapy, with some even showing exacerbation (28). Potential mechanisms of exacerbation include persistent tumor antigens after surgery (28), Th1/Th2 imbalance (29), surgical stress-induced inflammation (30), and chemotherapy-driven immune modulation (28). Due to the limited number of cases resulting from the low incidence of DM, more data need to be collected to enable more comprehensive investigations. On the other hand, bidirectional MR analysis can offer new insights from a genetic perspective, demonstrating a potential causal relationship between thyroid cancer and myositis but no significant association between myositis and thyroid cancer in the reverse direction. This unidirectional relationship aligns with the paraneoplastic hypothesis. However, the use of a myositis database, rather than a DM-specific dataset, limits the generalizability of our findings. Considering DM as the subtype most strongly associated with malignancy, the result tends to underestimate the true causal effect when DM is pooled together with other subtypes. A larger and more robust causal effect may be anticipated when a DM-specific dataset becomes available. No clinically relevant mutation was found in this patient’s WES. Further validation of genetic mutations is required in broader populations.

## Conclusion

DM accompanied by thyroid cancer may be more frequent than expected, with the underlying pathogenetic mechanisms remaining unclear. Previous studies have reported conflicting opinions regarding whether this association represents a paraneoplastic phenomenon or is unrelated. This article reports a DM patient with metastasis of PTC whose recurrence was indicated by the recurrence of the rash, which was resolved after surgical resection of metastatic foci, supporting the paraneoplastic hypothesis. MR analysis showed a potential causal relationship between myositis and thyroid cancer. Regarding the underlying mechanisms, an increased level of TIF-1γ protein in the tumor tissue of the DM patient supported the hypothesis that TIF-1γ was a tumor autoantigen released in large amounts from tumor tissue, thereby inducing autoantibody production and the development of DM. In the future, more data are needed to explore and confirm the relationship and pathogenetic mechanisms between thyroid cancer and DM.

## Data Availability

The original contributions presented in the study are included in the article/**Supplementary Material**. Further inquiries can be directed to the corresponding author.
