# Supplementary material for: The TIF-1γ autoantigen in patients with dermatomyositis and thyroid cancer: from case series to preliminary mechanistic exploration
Source: Front Immunol. 2026 Sep 8;17:1817558. doi: 10.3389/fimmu.2026.1817558 (PMC13593890; doi:10.3389/fimmu.2026.1817558)
Supplement: Supplementary file 1 [file Table1.docx]

**Supplementary table S1** The demographic characteristics of the dermatomyositis (DM) with thyroid cancer cases in the literature.

| Reference | Gender | Age at diagnosis | MSAs | CK level | ILD | Type of cancer | Temporal Relationship Between the Diagnoses | Anti-tumor therapy | Maintenance | Follow up |
| --- | --- | --- | --- | --- | --- | --- | --- | --- | --- | --- |
| Hatada et al^1^, 1996 | F | 50 | NA | NA | NA | PTC | Cancer diagnosis 4years before DM | Surgery | NA | NA |
| Fujita et al^2^, 2008 | F | 54 | NA | High | No | PTC | At the same time | Surgery with good response | NA | 1year without relapse |
| Nagashima et al^3^,  2009 | F | 59 | NA | High | Yes | PTC | At the same time | No | Low dose of glucocorticoid and immunosuppression therapy | 11months without relapse |
| Ikeda et al^4^, 2012 | F | 66 | NA | High | Yes | PTC | At the same time | Surgery with minimal response | NA | No relapse in 4 years |
| Lambova et al^5^, 2013 | F | 56 | NA | NA | NA | PTC | Cancer diagnosis 1years after DM | Surgery | NA | NA |
| Shah et al^6^, 2013 | M | 47 | NA | High | No | PTC | Cancer diagnosis 6months after DM | Improvement after thyroidectomy and radioiodine ablation | Low dose of glucocorticoid and immunosuppression therapy | 1year without relapse |
| Shah et al^6^, 2013 | F | 35 | NA | NA | NA | PTC | Cancer diagnosis 1year before DM | Surgery | glucocorticoid | 12years without relapse |
| Shah et al^6^, 2013 | F | 69 | NA | Normal | No | PTC | Cancer diagnosis 1year after DM | Surgery | NA | Lost |
| Molina-Ruiz et al^7^,  2015 | M | 53 | MDA5 | Normal | Yes | PTC | At the same time | Thyroidectomy and radioiodine ablation | NA | No improvement in 1year |
| Eranki^8^, 2017 | F | 62 | NA | NA | NA | PTC | Cancer diagnosis 3years after DM | Surgery with improvement | glucocorticoid | 4months without relapse |
| Yilmaz et al^9^,2021 | M | 35 | NA | High | Yes | PTC | At the same time | Thyroidectomy and radioiodine ablation | No | 2years without relapse |
| Legese et al^10^,2024 | F | 52 | NA | High | No | NA | At the same time | NA | glucocorticoid and immunosuppression therapy | Improvement after 2 months |
| Yang et al^11^,2025 | F | 60 | TIF1γ | High | No | PTC | Cancer diagnosis 8months before DM | Surgery | glucocorticoid and immunosuppression therapy | 30weeks without relapse |

Notes: MSAs, myositis specific autoantibodies; ILD interstitial lung disease; CK, creatine kinase; F, female; M, male; NA, not available; PTC, papillary thyroid carcinoma.

**Supplementary table S2** MR estimates and sensitivity analyses for associations of myositis and risk of thyroid cancer.

| nSNPs | Method | OR | 95CI% | Forest plot | P value | Q_P value |
| --- | --- | --- | --- | --- | --- | --- |
| 83 | MR-Egger | 1.005 | 0.984-1.027 |  | 0.628 | 0.86 |
| 83 | Weighted-median | 1.002 | 0.985-1.020 |  | 0.810 |  |
| 83 | Inverse variance weighted | 1.000 | 0.987-1.013 |  | 0.984 | 0.87 |
|  |  |  |  |  |  |  |

**Reference**

1.	Hatada T, Aoki I, Ikeda H, et al. Dermatomyositis and malignancy: case report and review of the Japanese literature. *Tumori* 1996; **82**(3): 273-5.

2.	Fujita M, Hatachi S, Yagita M. Dermatomyositis associated with thyroid cancer. *Rheumatol Int* 2008; **29**(2): 203-5.

3.	Nagashima T, Onishi S, Kamata Y, Minota S. Dermatomyositis associated with thyroid cancer: a paraneoplastic syndrome? *Rheumatol Int* 2009; **29**(10): 1261-2.

4.	Ikeda T, Kimura E, Hirano T, Uchino M. The association between dermatomyositis and papillary thyroid cancer: a case report. *Rheumatol Int* 2012; **32**(4): 959-61.

5.	Lambova S, Müller-Ladner U. Capillaroscopic pattern in paraneoplastic Raynaud's phenomenon. *Rheumatol Int* 2013; **33**(6): 1597-9.

6.	Shah M, Shah NB, Moder KG, Dean D. Three cases of dermatomyositis associated with papillary thyroid cancer. *Endocr Pract* 2013; **19**(6): e154-7.

7.	Molina-Ruiz AM, Romero F, Carrasco L, Feltes F, Haro R, Requena L. Amyophatic dermatomyositis presenting as a flagellated skin eruption with positive MDA5 antibodies and thyroid cancer: a real association? *Clin Exp Dermatol* 2015; **40**(8): 887-90.

8.	Eranki VG. A Recurrent Episode of Dermatomyositis Associated with Papillary Thyroid Cancer. *Case Rep Endocrinol* 2017; **2017**: 7985953.

9.	Yilmaz U, Ugurlu S. Dermatomyositis Associated With Papillary Thyroid Cancer: Systematic Review of the Literature and a Case Report. *J Clin Rheumatol* 2021; **27**(4): 168-71.

10.	Legese GL, Tesfaye YA, Ayele E, et al. Diagnosis of Thyroid Neoplasm-Associated Dermatomyositis in Ethiopian Woman. *Int Med Case Rep J* 2024; **17**: 201-7.

11.	Yang M, Liu J. Dermatomyositis following resection of thyroid and breast cancer: a case report and literature review. *Front Oncol* 2025; **15**: 1474481.
